# A Case of Solitary Nonvascularized Corneal Epithelial Dysplasia

**DOI:** 10.1155/2016/5687285

**Published:** 2016-03-03

**Authors:** Tomoya Morii, Takayoshi Sumioka, Ai Izutani-Kitano, Yukihisa Takada, Yuka Okada, Winston W.-Y. Kao, Shizuya Saika

**Affiliations:** ^1^Department of Ophthalmology, Wakayama Medical University School of Medicine, Wakayama, Japan; ^2^Department of Ophthalmology, University of Cincinnati School of Medicine, Cincinnati, OH, USA

## Abstract

*Background*. Epithelial dysplasia is categorized as conjunctival/corneal intraepithelial neoplasia which is a precancerous lesion. The lesion is usually developed at the limbal region and grows towards central cornea in association with neovascularization into the lesion. Here, we report a case of isolated nonvascularized corneal epithelial dysplasia surrounded by normal corneal epithelium with immune histochemical finding of ocular surface tissues cytokeratins, for example, keratin 13 and keratin 12.* Case Presentation*. A 76-year-old man consulted us for visual disturbance with localized opacification of the corneal epithelium in his left eye. His visual acuity was 20/20 and 20/200 in his right and left eye, respectively. Slit lamp examination showed a whitish plaque-like lesion at the center of his left corneal epithelium. No vascular invasion to the lesion was found. The lesion was surgically removed and subjected to histopathological examination and diagnosed as epithelial dysplasia. Amyloidosis was excluded by direct fast scarlet 4BS (DFS) staining. Immunohistochemistry showed that the dysplastic epithelial cells express keratin 13 and vimentin, but not keratin 12, indicating that the neoplastic epithelial cells lacked corneal-type epithelium differentiation.* Conclusions*. The lesion was diagnosed as nonvascularized epithelial dysplasia of ocular surface. Etiology of the lesion is not known.

## 1. Introduction

Epithelial dysplasia is categorized as conjunctival/corneal intraepithelial neoplasia, which is a precancerous lesion [1]. Corneal epithelial dysplasia occurs in elder ages. Although the etiology remains unknown, it has been suggested that excess ultraviolet exposure and infection of human papilloma virus may cause the lesion [2, 3]. The pathogenesis of corneal epithelial neoplasia usually commences at the limbal region in that abnormal epithelium grows towards central cornea and often associates with neovascularization into the lesion. Here we report a case of isolated nonvascularized corneal epithelial dysplasia surrounded by normal corneal epithelium with immunohistochemical finding.

## 2. Case Presentation

**Figure 1:**
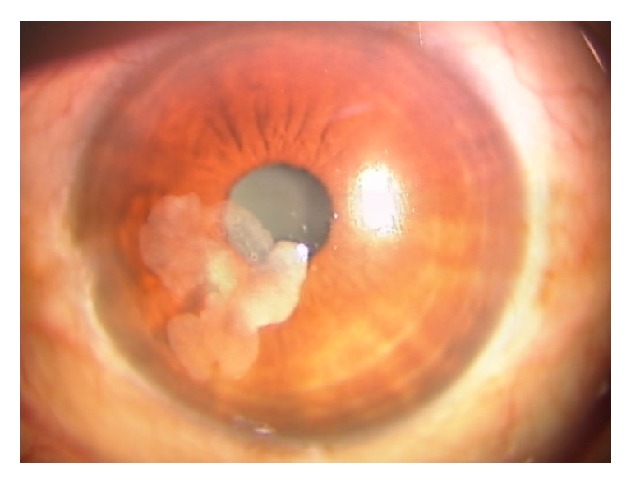
Avascular whitish plaque-like lesion is observed in the cornea.

**Figure 2:**
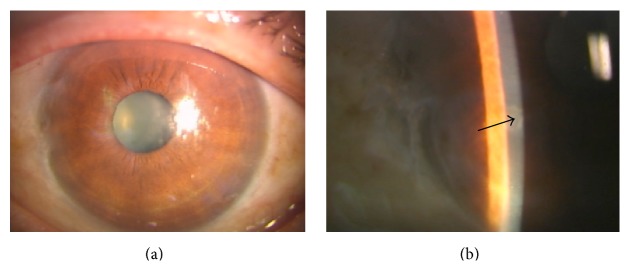
(a) One month after the removal of the lesion. The lesion did not recur. (b) Stroma beneath the original tumor is somewhat opaque (arrow).

A 76-year-old man consulted us for visual disturbance with corneal opacity in his left eye (Figure 1). He had received topical medication for chronic open-angle glaucoma for over 5 years. We did not obtain significant information on the etiologic factors related to neoplasm induction. His visual acuity was 20/20 and 20/200 in the right and left eye, respectively. Intraocular pressure (IOP) in his right eye was 14 mmHg, but IOP could not be precisely measured in his left eye due to the elevated lesion on the corneal surface. A whitish elevated, plaque-like isolated lesion without neovascularization was observed in the central area of his left cornea. The cornea of his right eye did not show any abnormality or signs of intraocular inflammation. The whitish plaque-like lesion was removed under topical anesthesia. The lesion was easily peeled off from the underlying basement membrane. The epithelial defect produced by the removal of the lesion was resurfaced by normal-looking epithelium in a couple of days. No recurrent lesion was observed after seven months of surgery (Figure 2(a)). Stroma beneath the neoplasm is opaque (Figure 2(b)). The removed lesion epithelium was fixed, paraffin-embedded, and subjected to histology/immunohistochemistry examinations with hematoxylin-eosin (HE) staining, direct fast scarlet 4BS (DFS) staining, and immunohistochemistry for intermediate filaments of mesenchyme and ocular surface epithelial cells such as vimentin, keratin 12, keratin 13, and keratin 14, as previously described [4].

**Figure 3:**
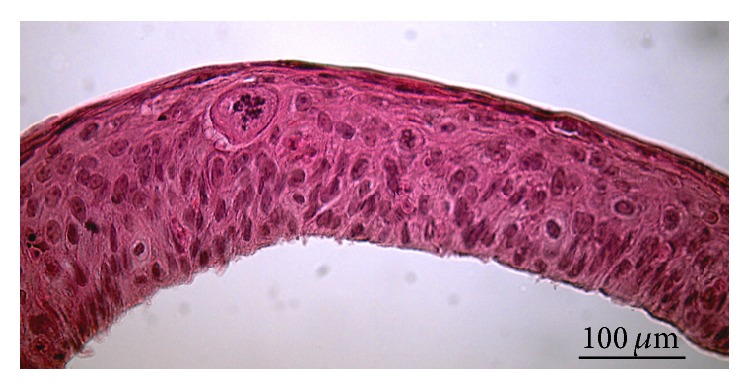
Hematoxylin-eosin (HE) staining shows that the epithelium is occupied with dysplastic cells with differentiation tendency from basal layer toward superficial layer.

**Figure 4:**
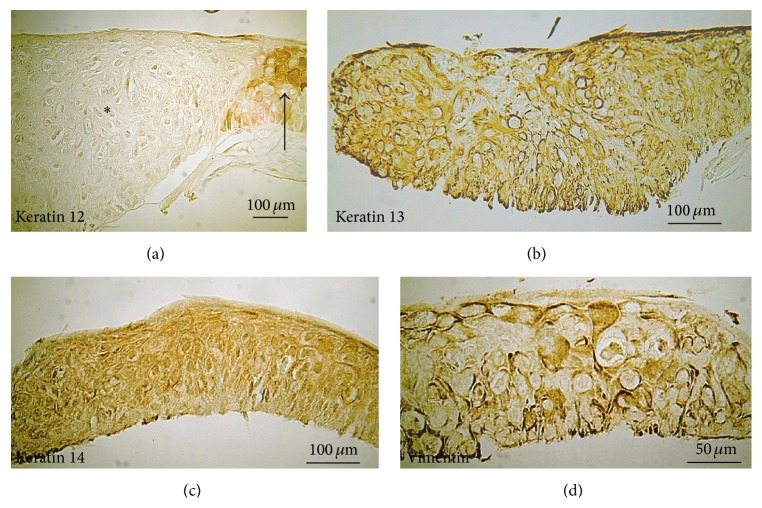
Immunohistochemical detection of intermediate filament components in dysplastic epithelium. Keratin 12 (a) is not expressed in dysplastic epithelial cells (asterisk), while it is well detected in normal corneal epithelium (arrow) adjacent to the lesion. Keratin 13 (b) and keratin 14 (c) are readily detected in the dysplastic cells throughout the layers. Some of the dysplastic cells are labeled for vimentin (d).

The epithelium of the lesion was occupied with atypical dysplastic epithelial cells as revealed by HE staining (Figure 3). The specimen was not labeled for DFS staining and thus the lesion was not related to an amyloid disease. Immunohistochemistry of cell lineage-specific intermediate filaments, for example, vimentin of mesenchymal cells, keratin 14 of a basal cell of stratified epithelium, keratin 12 of corneal epithelium, and keratin 13 of conjunctival epithelium, showed that the dysplastic cells were positively labeled for vimentin, keratin 13, and keratin 14, but not for keratin 12 (Figure 4). Expression of Krt14 was detected in all epithelial cell layers through basal to superficial layers, a phenotype frequently seen in pathogenic hyperplasia of stratified epithelium including cornea and epidermal epithelium [5]. Many cells in the lesion are also positively labeled by Krt13 and vimentin implicating that these cells could undergo transdifferentiation from corneal to conjunctival epithelial cells or could be directly derived from conjunctival epithelium. The presence of vimentin strongly implicates the possibility that transdifferentiation might have taken place in the formation of the abnormal epithelium seen in the lesion.

## 3. Discussion

We report here a rare case of isolated dysplasia of the corneal epithelium that was not associated with neovascularization. The lesion resembled corneal epithelial amyloidosis that was excluded by a finding of negative DFS staining. Histology examination was critical to the precise diagnosis in the present case and indicated the diagnosis of isolated nonvascularized tumor lesion of corneal epithelial dysplasia but not amyloidosis. Immunohistochemistry revealed that the dysplastic cells are positive for vimentin and keratin 14, thus underlying the process of dedifferentiation and/or epithelium-mesenchymal transition, a characteristic of neoplastic epithelial cell type [6]. Lack of keratin 12 and marked keratin 13 expression also supports the notion that the dysplastic cells lost corneal-type epithelial differentiation rather than the invasion and/or differentiation of conjunctival epithelial cells.

Usually, ocular surface epithelial dysplasia occurs at or around the limbal region, which gradually invades into corneal and/or conjunctival epithelium and is often accompanied by neovascularization. The present case, however, showed two unusual characteristics: (1) isolated lesion surrounded by normal corneal epithelium and (2) lack of neovascularization. Corneal stem cells reside in limbus, which continuously replenish corneal epithelium. The healthy limbal epithelium is critical for avascularity and optically smooth-surfaced epithelium of the cornea. In the central/bottom region of the limbal crypt a horizontal asymmetric cell division of the stem cell produces another stem cell. Progenitor cells at the border in between limbus and cornea may produce transient amplifying basal cell that assumes pericentral migration from the limbal region by yielding the space to symmetric and/or asymmetric cell division of a neighboring transient amplifying basal cell. On the other hand, limbal stem cell produces limbal upward cells by vertical asymmetric cell division and produces another stem cell by symmetric cell division inside the limbus [7, 8]. Usually ocular surface neoplasm occurs at the site of limbus and spreads to both corneal epithelium and conjunctival epithelium. The origin of this type of neoplasm is considered to be the stem cell residing in the limbal crypt of Vogt. On the other hand, in the present case, the neoplastic lesion was surrounded by the normal corneal epithelium and thus was considered to migrate along with the centripetal movement of corneal epithelial sheet. The origin of the lesion of the current case might be derived from a limbal stem/progenitor cell that failed to completely assume corneal type of epithelial cells differentiation.
